# Genome-Wide Profiling of H3K27ac in Tianzhu White Yak Skin at the Anagen and Telogen Stages

**DOI:** 10.3390/biology15211920

**Published:** 2026-10-23

**Authors:** Lanlan Cai, Tianfen Guo, Qingbo Zheng, Min Chu, Xiaoming Ma, Xian Guo, Pengjia Bao, Chunnian Liang, Xiaoyun Wu

**Affiliations:** Key Laboratory of Animal Genetics and Breeding on Tibetan Plateau, Ministry of Agriculture and Rural Affairs, Key Laboratory of Yak Breeding Engineering, Lanzhou Institute of Husbandry and Pharmaceutical Sciences, Chinese Academy of Agricultural Sciences, Lanzhou 730050, China; cailanlan007@163.com (L.C.);

**Keywords:** yak, hair follicle, H3K27ac, acetylation, super-enhancer

## Abstract

The Tianzhu white yak, native to Gansu Province, China, is valued for its white coat and fibre traits. Its hair follicles undergo pronounced seasonal cycling, but the associated epigenomic regulation remains poorly understood. We profiled H3K27ac in skin samples collected during anagen and telogen using Cleavage Under Targets and Tagmentation (CUT&Tag) and integrated these data with RNA sequencing. We identified 14,496 differentially enriched regions, including 5618 with increased and 8878 with decreased H3K27ac enrichment during anagen. Associated genes were enriched in pathways governing follicle activation, epithelial remodelling and tissue homeostasis, including Wnt, PI3K-AKT, MAPK and TGF-beta signalling. Putative targets of anagen-specific super-enhancers were enriched in PI3K-AKT and Notch pathways. Motif analysis revealed enrichment of binding motifs for transcription factors implicated in hair follicle biology, including LEF1, LHX2, FOXN1 and GATA3, whereas FOSL2 and FOS::JUNB motifs were preferentially enriched in anagen-gain regions. Integrative CUT&Tag and RNA-seq analysis identified 765 genes showing concordant changes in H3K27ac enrichment and transcript abundance between anagen and telogen, including candidate hair follicle regulators such as *FGF5*, *PDGFRA*, *TCF7* and *SMAD7*. These findings characterize phase-associated regulatory landscapes and identify candidate regulatory elements and genes potentially involved in hair cycling and fibre traits.

## Simple Summary

The Tianzhu white yak is a unique yak breed known for its pure white coat and valuable fibre. Its hair follicles undergo seasonal cycles between an active-growth phase, known as anagen, and a resting phase, known as telogen. However, changes in gene expression levels and regulatory regions between these two phases remain unclear. We compared skin samples collected during these two phases and examined both gene activity and the regions of DNA that regulate it. We identified extensive differences in these regulatory regions between the anagen and telogen phases. Genes associated with these regions were involved in hair follicle activation, tissue remodelling and homeostasis. The two phases also showed distinct super-enhancer and transcription-factor binding motifs that may contribute to changes in gene activity during the hair cycle. By combining information on gene-regulatory activity and gene expression, we identified candidate genes that may help regulate seasonal hair growth. These findings improve our understanding of the molecular regulation of hair cycling and provide a resource for future studies of hair growth and fibre-related traits in Tianzhu white yaks.

## 1. Introduction

Tianzhu white yak (*Bos grunniens*) is a distinctive breed distributed mainly in Tianzhu Tibetan Autonomous County, Gansu Province, China, and is listed as a protected genetic resource in China’s National Livestock and Poultry Genetic Resources. The breed is valued for both meat and fibre production and is distinguished by its uniformly white coat. Its fleece comprises two major fibre types: coarse guard fibres forming the outer coat and fine down fibres constituting the undercoat [1]. Owing to its natural white color, high dyeability, and favorable processing properties, Tianzhu white yak fibre has considerable economic value. Accordingly, improving fibre-related traits is an important objective of breeding programs for this breed.

Fibre yield and quality are largely determined by hair follicles (HFs), highly specialized cutaneous mini-organs in fibre-producing mammals [2,3]. In yaks, hair HFs are classified as primary follicles, which produce coarse guard fibres in the outer coat, and secondary follicles, which generate fine down fibres in the undercoat [1]. These follicles undergo a seasonal growth cycle comprising anagen, catagen, and telogen phases; their cyclic activation and regression directly influence fibre growth, yield, and quality [4]. Previous studies have shown that HF cycling is regulated by a coordinated network of signaling pathways, including Wnt/β-catenin [5], fibroblast growth factor (FGF) [6], sonic hedgehog (SHH) [7], bone morphogenetic protein (BMP) [8], and Notch [9], which collectively control follicle activation, regression, and quiescence. More recently, bulk and single-cell transcriptomic analyses have provided valuable insights into the molecular mechanisms underlying yak HF development and cycling, and have identified numerous candidate genes associated with follicle morphogenesis and seasonal growth [4,10,11,12].

In addition to transcriptional regulation, epigenetic modifications play crucial roles in controlling gene expression [13]. Histone acetylation is commonly associated with increased chromatin accessibility; it reduces histone–DNA affinity and facilitates the transition of chromatin from a compacted state to a more relaxed conformation, thereby enabling transcription factors and other regulatory proteins to bind [14]. Among histone modifications, Histone H3 lysine 27 acetylation (H3K27ac) is widely recognized as an epigenetic marker of open chromatin and transcriptional activation [15]. Specifically, H3K27ac marks active promoters and enhancers, which are key *cis*-regulatory elements mediating gene expression. Promoters initiate transcription by recruiting RNA polymerase and general transcription factors, while enhancers regulate transcription from a distance via chromatin looping interactions between enhancers and promoters [16,17]. Enrichment of H3K27ac at these regulatory elements is broadly interpreted as a hallmark of transcriptionally active states and functional regulatory activity.

Super-enhancers (SEs), which consist of dense clusters of individual enhancers, are characterized by exceptionally high levels of transcription factor binding, coactivator recruitment, and H3K27ac signals. Compared with typical enhancers, SEs preferentially regulate critical genes associated with cell identity and differentiation-related biological processes [18]. Genome-wide analyses of H3K27ac in livestock species have demonstrated that enhancers play essential roles in organ development and disease progression [19,20]. However, the genome-wide dynamics of H3K27ac during different phases of the yak HF cycle, as well as their interplay with transcriptional regulation, remain unexplored.

In this study, we characterized H3K27ac modifications in the skin tissues of Tianzhu white yak during the anagen and telogen phases using Cleavage Under Targets and Tagmentation (CUT&Tag). By integrating CUT&Tag data with transcriptome profiles, we elucidated the relationship between H3K27ac modifications and gene expression, and identified potential SEs associated with stage-specific regulatory programs. These findings offer novel insights into the epigenetic regulation of HF development in yak and provide potential candidate regulatory elements and genes potentially contributing to hair cycling and fibre traits.

## 2. Materials and Methods

### 2.1. Samples Collection

The experimental Tianzhu white yaks were obtained from local farmers in Tianzhu County, Gansu Province, China. Six healthy three-year-old female yaks were selected. Skin biopsies were collected from the right scapular region of yaks. The anagen and telogen groups each comprised three different animals (*n* = 3 biological replicates per phase), and no individual represented in both groups. Prior to sampling, the hair over the scapular region was clipped with scissors, and the sampling site was aseptically prepared with 75% ethanol. Local anaesthesia was induced by subcutaneous injection of a total of 5 mL of 2% lidocaine at the designated biopsy site. Approximately 2 cm^2^ of skin was then excised using sterile surgical scissors. The wounds were closed with absorbable sutures and disinfected with povidone-iodine. The yaks were monitored postoperatively for their general health and retained for breeding. The excised skin samples were immediately snap-frozen in liquid nitrogen for preservation. Samples from the late-anagen phase, representing the late stage of the anagen phase of the hair cycle, were collected on 17 October 2025, whereas telogen-phase samples were collected on 8 May 2026.

### 2.2. H3K27ac CUT&Tag

An H3K27ac-targeted CUT&Tag assay was conducted to profile genome-wide chromatin-associated H3K27ac signals, as described previously [21]. Skin samples were snap-frozen and ground into fine powder using liquid nitrogen, followed by the addition of lysis buffer to ensure complete tissue disruption. Activated Concanavalin A-coated magnetic beads (ConA Beads) were incubated with pooled nuclei in Binding Buffer at room temperature for 10 min. Nuclei bo6und to beads were incubated with the anti-H3K27ac primary antibody (Cell Signaling Technology, Danvers, MA, USA; 8173S, RRID: AB_10949503, 1:50 in Dig-Wash Buffer) for 2 h at room temperature, followed by incubation with affinity-purified polyclonal goat anti-rabbit IgG antibody (Solarbio, Beijing, China; SPA134, RRID: not available, 1:100 in Dig-Wash Buffer) for 1 h at room temperature. The pG-Tn5 adapter complex was diluted 1:200 in Dig-300 buffer to a final concentration of approximately 0.04 μM. Dig-300 buffer consisted of 0.01% digitonin, 20 mM HEPES (pH 7.5), 300 mM NaCl, 0.5 mM spermidine, and 1 × protease inhibitor cocktail. For each sample, 100 μL of the diluted pG-Tn5 preparation was added and incubated for 1 h. Unbound pG-Tn5 was removed by washing the bead-bound nuclei twice with 500 μL of Dig-300 buffer for 1 min per wash. The nuclei were subsequently resuspended in 300 μL of Tagmentation buffer (10 mM MgCl_2_ in Dig-300 Buffer), and incubated at 37 °C for 1 h. Immediately following tagmentation, the reaction was terminated by adding a stop solution consisting of 10 μL 0.5 M EDTA, 3 μL 10% SDS, and 2.5 μL 20 mg/mL Proteinase K. DNA extraction was performed using the phenol/chloroform method, followed by ethanol precipitation. The extracted DNA fragments were amplified for 16 cycles, purified with AMPure XP beads and subsequently sequenced on the Illumina NovaSeq platform (Illumina Inc., San Diego, CA, USA), producing 150 bp paired-end reads.

### 2.3. CUT&Tag Data Analysis

Raw sequencing reads were processed for quality control using Trimmomatic (v0.39) [22], which removed adapter contamination and low-quality nucleotide sequences. The filtered reads were then aligned to the yak reference genome (LU_Bosgru_v3.0) [23] using Bowtie2 v2.2.9 [24]. PCR duplicates were subsequently removed using Picard MarkDuplicates (v2.25.6). An IgG antibody control was not included in the CUT&Tag reaction. Peak calling was performed using MACS2 software (v2.2.9.1) [25] with the parameters: -f BAMPE -B --SPMR --keep-dup all. Visualization of the data was achieved by generating BigWig files using deepTools (v3.5.1) [26]. Differentially enriched peak analysis was carried out on the consensus peakset using the DiffBind R package (v3.2.6) [27] coupled with the DESeq2 method. The default DESeq2 workflow was used for count normalization, size factor estimation, dispersion modeling, and hypothesis testing. Peaks were considered significantly different with thresholds of |log2FC| > 0.58 and false discovery rate (FDR) < 0.05. Genes associated with differentially enriched peaks were annotated using the ChIPseeker R package (v1.20.0) [28], by assigning peaks to the nearest transcription start site (TSS).

### 2.4. Motif Analyses

Motif enrichment analysis of differential enriched peaks was performed using AME [29], in the MEME Suite (v5.4.1) [30], with the merged peaks across all samples were used as the background. Motifs with *p* values < 0.05 were considered significant.

### 2.5. Super-Enhancer Identification

SEs were identified using the ROSE (Rank Ordering of SEs) algorithm according to the methodology established by the Young Laboratory [18,31]. In this approach, ROSE merges peak regions located within 12.5 kb of each other, calculates normalized tag counts (alignment reads derived from the BED file) for the combined regions, and assigns ranks based on tag counts. A tangent line with a slope of 1 was used as the cutoff to identify SEs. Enhancer regions located above the tangent line were classified as SEs.

### 2.6. RNA-Seq Data Processing

The RNA-seq dataset was generated from aliquots of the same skin specimens used for the CUT&Tag experiments. Raw sequencing reads were processed with SOAPnuke v1.5.2 [32], ensuring high-quality sequences, which were subsequently aligned to the yak reference genome (LU_Bosgru_v3.0) [23] using HISAT2 v2.1.0 [33]. Transcript reconstruction was performed using StringTie (v1.3.5), and the assembled transcripts were utilized as references for accurate quantification. Clean reads were realigned to the assembled transcripts using Bowtie2 (v2.2.9) [24], and transcript abundances were calculated as FPKM (Fragments Per Kilobase of transcript per Million mapped reads) values using RSEM (v1.3.1) [34]. Raw counts for each sample were used for gene differential expression analysis with DESeq2 (v1.20.0) [35]. Genes were considered significantly differentially expressed if they satisfied the thresholds of a false discovery rate (FDR) < 0.05 and an absolute log_2_fold change > 1.

### 2.7. Functional Annotation and Pathway Analysis

Gene Ontology (GO) and Kyoto Encyclopedia of Genes and Genomes (KEGG) pathway enrichment analysis was performed using the hypergeometric test with DAVID tools (https://davidbioinformatics.nih.gov/, accessed on 22 July 2026) [36], with all annotated genes in the *Bos taurus* genome used as the background. GO terms and KEGG pathways with an FDR of less than 0.05 were deemed significantly enriched.

### 2.8. CUT&Tag and RNA-Seq Overlap Analysis

Genes associated with H3K27ac peaks were ranked by the signal intensity of their respective peaks and then separated into three groups of equal size (tertiles). These tertiles were designated low, middle, and high according to their peak-signal levels. Normalized transcript abundances were then compared among the three groups using Kruskal–Wallis test. In addition, genes associated with differential H3K27ac peaks were overlapped with differentially expressed genes (DEGs). Only DEGs associated with at least one H3K27ac peak were included in the overlap analysis. Fisher’s exact test was used to determine whether the overlap was statistically significant.

## 3. Results

### 3.1. Quality Assessment and Genome-Wide Characterization of H3K27ac CUT&Tag Profiles in Yak Skin

**Figure 1:**
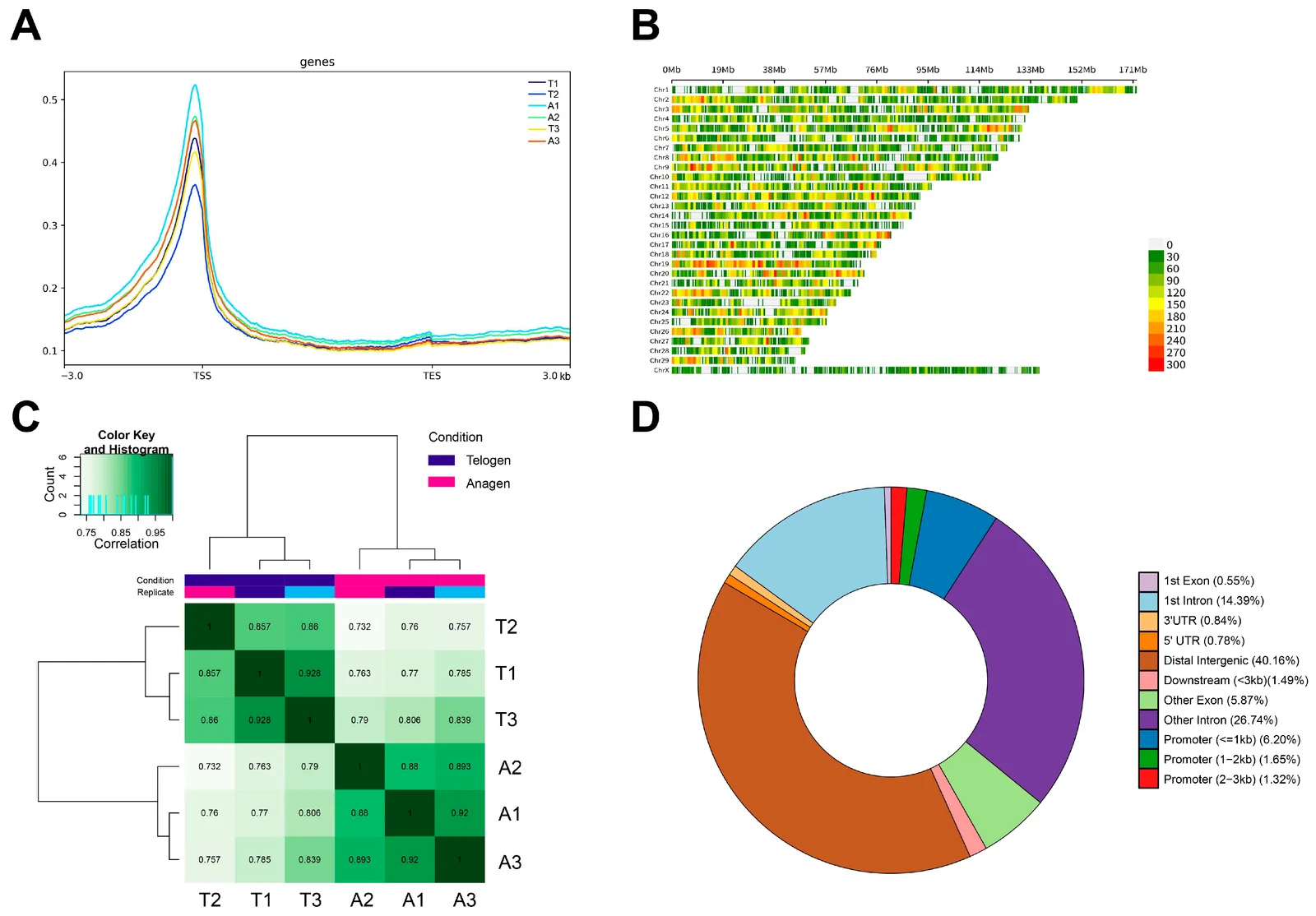
Genome-wide region of H3K27ac in yak skin during anagen and telogen. (**A**) Enrichment of H3K27ac reads across gene bodies. Each line represents one sample. (**B**) Genome-wide distribution of H3K27ac peaks in the yak genome. (**C**) Correlation heatmap showing the reproducibility among biological replicates. (**D**) Genomic annotation of H3K27ac peaks identified in the anagen and telogen groups. A, anagen; T, telogen.

H3K27ac CUT&Tag sequencing was performed on skin samples from yaks in the anagen (*n* = 3) and telogen (*n* = 3) phases of the HF cycle. Each sample yielded an average of 11.64 Gb of raw sequencing data. Following adapter trimming and quality filtering, more than 99.00% of reads were retained as clean reads. Q20 and Q30 values ranged from 98.71% to 99.58% and from 92.86% to 97.30%, respectively. The overall mapping rates ranged from 96.32% to 96.66%, while the fraction of reads in peaks (FRiP scores) ranged from 0.23 to 0.48 (Table S1). To characterize the genomic distribution of H3K27ac, signal profiles were generated within ±3 kb of the TSSs and transcription end sites (TESs). H3K27ac signal was enriched near the TSSs and progressively decreased across gene bodies (Figure 1A). A mean of 104,069 peaks was identified per sample, with the number ranging from 74,240 to 162,645. Merging peak calls across samples yielded a nonredundant peak set distributed across most autosomes (Figure 1B). Principal component analysis (PCA) (Figure S1) and correlation heatmaps further showed high similarity among biological replicates within each phase (Figure 1C). Collectively, these results support the quality and reproducibility of the dataset for downstream analyses.

### 3.2. Differential H3K27ac Enrichment Is Associated with Stage-Specific Regulatory Programs in Yak Skin

To investigate dynamic changes in H3K27ac enrichment between distinct stages of the HF cycle, we compared genome-wide H3K27ac profiles between anagen and telogen skin samples. Genomic annotation showed that H3K27ac peaks were predominantly located in distal intergenic regions, introns, and promoters (≤1 kb from the TSS) (Figure 1D; Table S2), indicating that H3K27ac marks both promoter-proximal and distal regulatory elements in yak skin.

**Figure 2:**
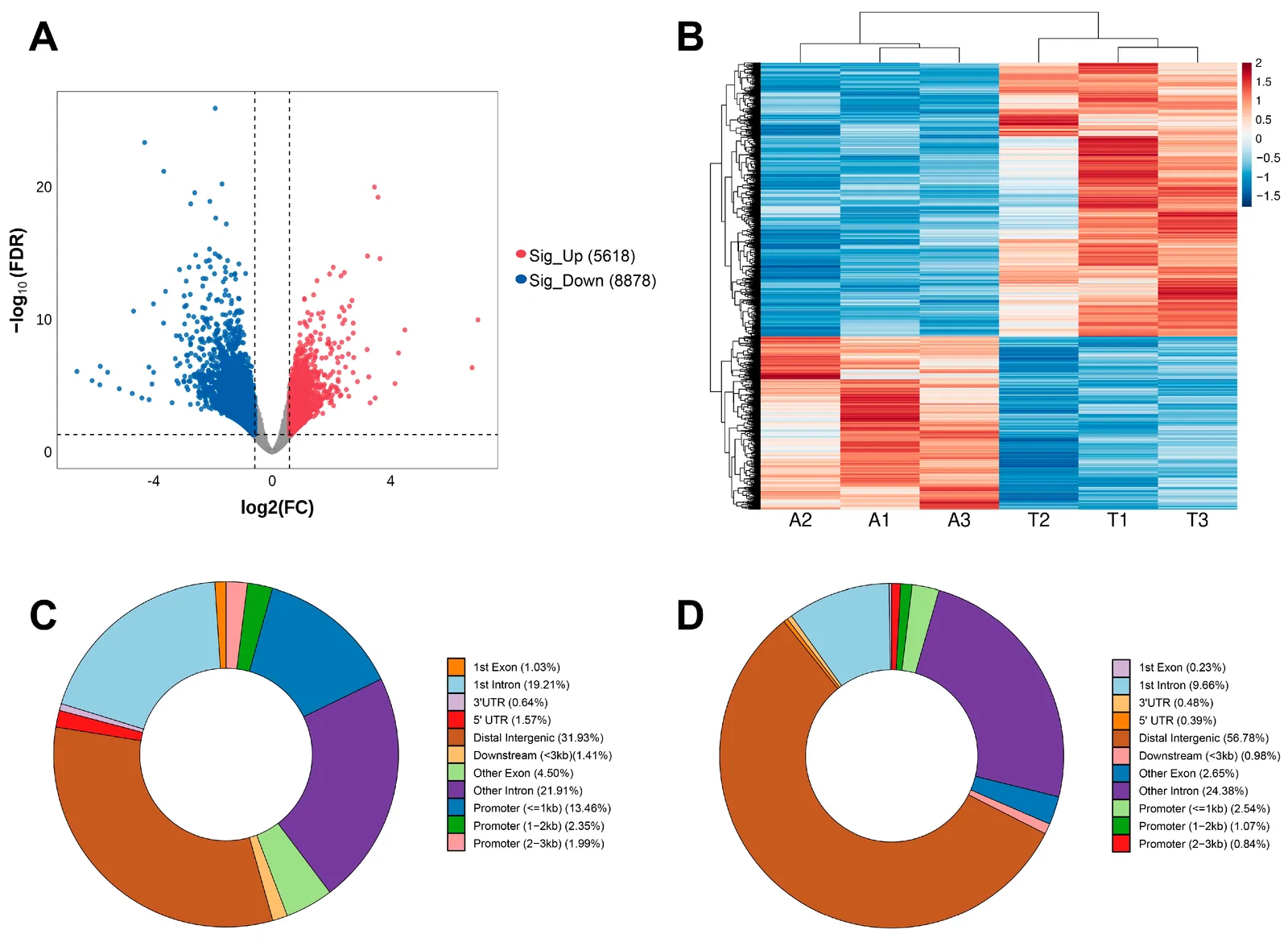
Differential H3K27ac enrichment in yak skin between anagen and telogen. (**A**) Volcano plot showing differentially enriched H3K27ac peaks. (**B**) Heatmap showing the signal intensity of differentially enriched H3K27ac peaks in anagen and telogen groups. (**C**) Genomic distribution of H3K27ac-gain peaks during anagen. (**D**) Genomic distribution of H3K27ac-loss peaks during anagen.

We identified 14,496 differentially enriched H3K27ac peaks between anagen and telogen, including 5618 peaks with increased enrichment during anagen (hereafter, H3K27ac-gain peaks) and 8878 peaks with decreased enrichment during anagen (hereafter, H3K27ac-loss peaks). Details and visualizations are provided in Figure 2A,B and Table S3. H3K27ac-gain peaks were preferentially located in introns (41.12%) and distal intergenic regions (31.93%) (Figure 2C), whereas H3K27ac-loss peaks had a higher proportion of distal intergenic regions (56.78%) and introns (34.04%) (Figure 2D). These distinct genomic distributions suggest that stage-associated changes in H3K27ac may involve partially distinct regulatory architectures.

**Figure 3:**
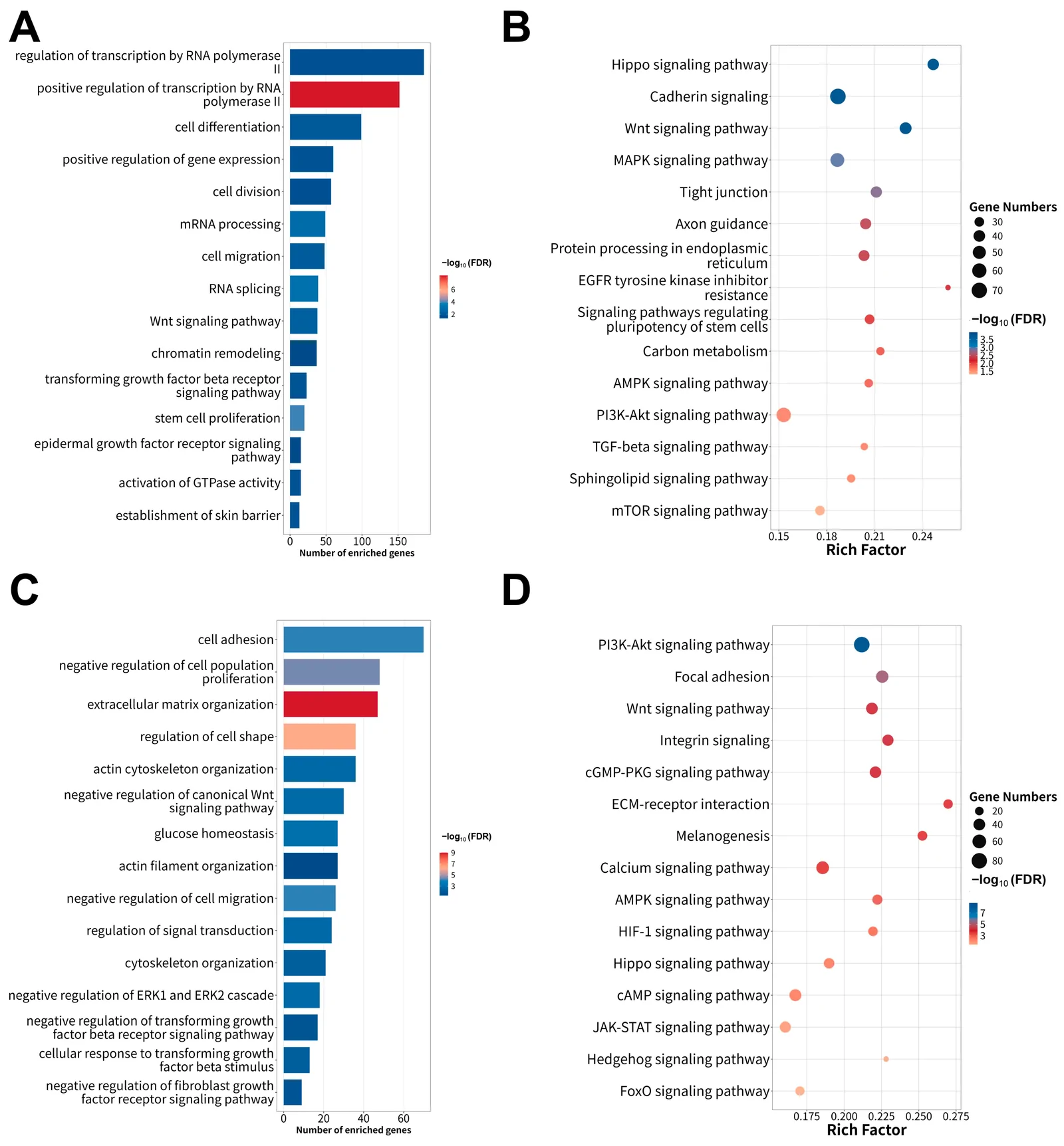
Functional enrichment analysis of genes associated with differentially enriched H3K27ac peaks in yak skin between anagen and telogen. (**A**) GO enrichment analysis of genes associated with H3K27ac-gain peaks during anagen. (**B**) KEGG pathway enrichment analysis of genes associated with H3K27ac-gain peaks during anagen. (**C**) GO enrichment analysis of genes associated with H3K27ac-loss peaks during anagen. (**D**) KEGG pathway enrichment analysis of genes associated with H3K27ac-loss peaks during anagen.

To assign candidate target genes, each differentially enriched peak was linked to the nearest TSS within a 100 Kb window. In total, 6873 genes were associated with the differentially enriched H3K27ac peaks. Genes associated with anagen H3K27ac-gain peaks were enriched for biological processes related to HF activation and cellular proliferation, including stem cell proliferation, establishment of the skin barrier, cell division, and epidermal growth factor receptor signalling pathway (Figure 3A; Table S4). These genes were also enriched in KEGG pathways involved in HF growth and epithelial remodelling, including the Hippo, cadherin, Wnt, MAPK, PI3K-AKT, and TGF-beta signalling pathways (Figure 3B; Table S5).

By contrast, genes associated with anagen H3K27ac-loss peaks (and therefore exhibiting relatively higher H3K27ac enrichment in telogen) were enriched for biological processes related to HF quiescence and tissue homeostasis including extracellular matrix organization, negative regulation of cell population proliferation, cell adhesion, and negative regulation of fibroblast growth factor receptor signalling pathway (Figure 3C; Table S6). These genes were also enriched in signalling pathways implicated in HF homeostasis, including focal adhesion, ECM–receptor interaction, JAK–STAT, Hedgehog, calcium, FoxO, and AMPK signalling pathways (Figure 3D; Table S7).

**Figure 4:**
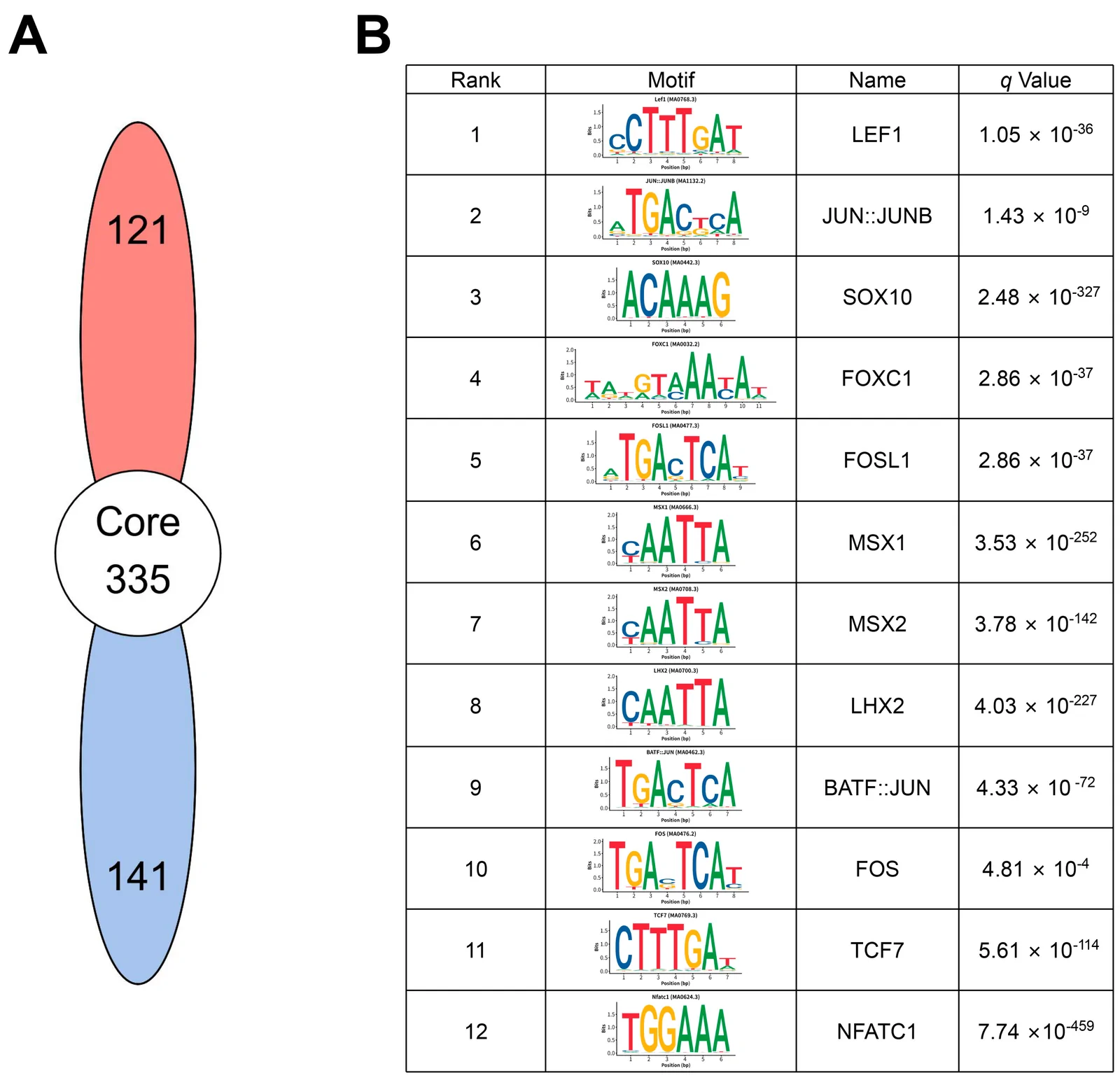
Motif enrichment analysis of differentially enriched H3K27ac peaks. (**A**) Shared transcription factors associated with H3K27ac gain and loss peaks. (**B**) Motif enrichment analysis of differentially enriched H3K27ac peaks associated with HF development. The sequence logo represents the identified motif, where letter heights indicate base conservation and colors represent specific nucleotides (green: A, blue: C, yellow: G, red: T).

To identify transcription factors (TFs) potentially associated with differentially enriched H3K27ac peaks in anagen and telogen, we performed TF motif-enrichment analyses using proximal and distal peak sequences. Most enriched motifs were shared between anagen-gain and anagen-loss peaks (Figure 4A; Table S8), including motifs associated with the MSX family (MSX1 and MSX2), the TCF/LEF family (LEF1, TCF7L2, TCF7, and TCF3), the Forkhead family (FOXN1, FOXC1, and FOXD1), AP-1 and bZIP factors (BATF::JUN, JUN::JUNB, FOS::JUNB, FOS, FOSL1, and FOSL2), LHX2 and NFATC1 (Figure 4B). Notably, FOSL2 and FOS::JUNB motifs were specifically enriched in anagen-gain peaks, suggesting that AP-1-mediated transcriptional regulation may contribute specifically to the activation of the anagen-associated H3K27ac landscape during the telogen-to-anagen transition and in the activation of regulatory elements linked to HF regeneration.

### 3.3. Genome-Wide Identification of H3K27ac-Associated Super-Enhancers in Anagen and Telogen Skin

**Figure 5:**
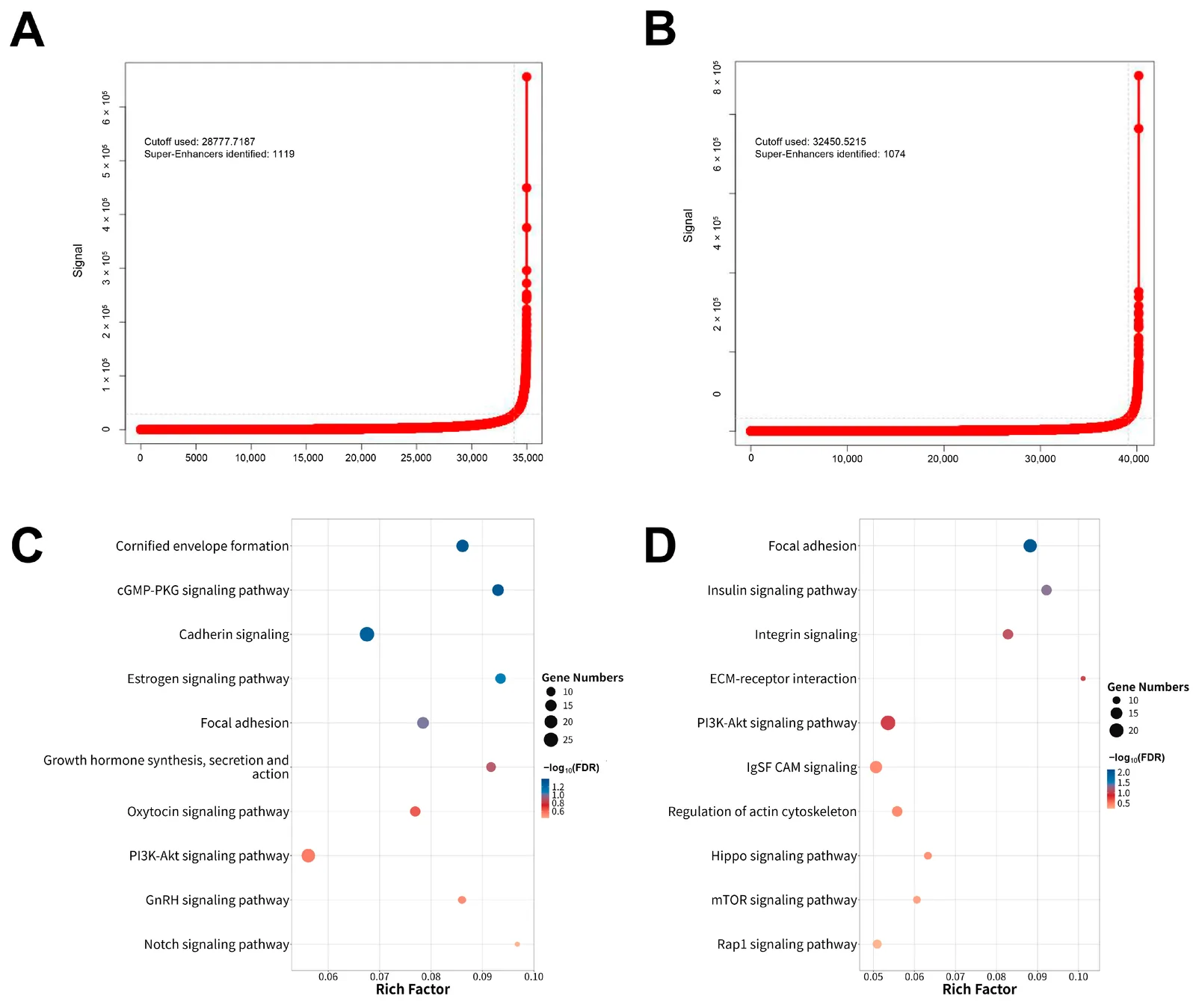
Identification of H3K27ac-based SEs in the skin during anagen and telogen. (**A**,**B**) Ranking of H3K27ac signal between anagen and telogen phase. (**C**) KEGG pathway enrichment analysis of putative target genes of anagen-specific SE. (**D**) KEGG pathway enrichment analysis of putative target genes of telogen-specific SE.

To further characterize stage-specific regulatory landscapes, we examined H3K27ac-marked SEs. SEs were identified using the ROSE algorithm, which ranks enhancer regions according to their H3K27ac signal intensity. We identified 1119 SEs in anagen (Figure 5A; Table S9) and 1074 in telogen (Figure 5B; Table S10), of which 310 were anagen-specific and 304 were telogen-specific. Functional enrichment analysis showed that the putative target genes of anagen-specific SEs were enriched in biological processes, including stem cell proliferation, keratinocyte differentiation, and cell division (Table S11). By contrast, putative target genes of telogen-specific SEs were enriched in biological processes, including regulation of cell cycle and negative regulation of transforming growth factor beta receptor signaling pathway (Table S12). KEGG pathway analysis showed that putative target genes of anagen-specific SEs were primarily involved in the PI3K-AKT and Notch signalling pathways, as well as growth hormone production, secretion, and activity (Figure 5C; Table S13). Putative target genes of telogen-specific SEs were enriched in the focal adhesion, ECM–receptor interaction, and Rap1 signalling pathways (Figure 5D; Table S14). These findings suggest that SEs contribute to stage-specific regulation of gene expression between the anagen and telogen phases of the HF cycle.

### 3.4. Transcriptomic Profiling Reveals Gene Expression Dynamics During HF Cycle

**Figure 6:**
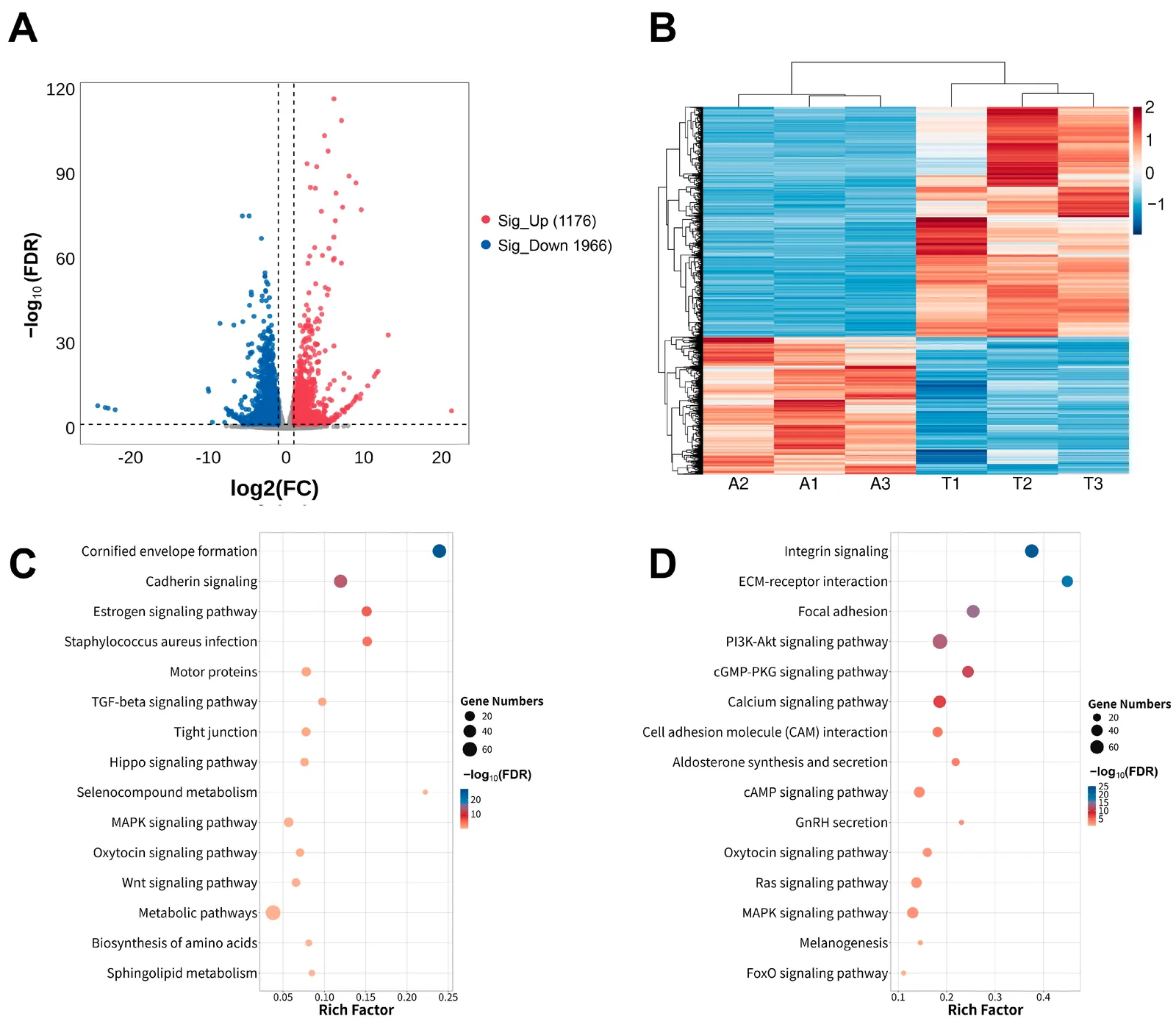
Transcriptomic profiling of yak skin during anagen and telogen. (**A**) Volcano plot of differentially expressed genes (FDR < 0.05). (**B**) Heatmap showing the expression patterns of DEGs in anagen and telogen samples. (**C**) KEGG pathway enrichment analysis of genes upregulated during anagen. (**D**) KEGG pathway enrichment analysis of genes downregulated during anagen.

In total, 24,406 mRNAs were detected, including 3142 DEGs in yak skin. Of these DEGs, 1176 were upregulated and 1966 were downregulated in anagen relative to telogen (Figure 6A,B; Table S15). Functional enrichment analysis of all DEGs identified biological processes related to follicle formation and tissue remodelling, including establishment of skin barrier, cell differentiation, epithelial cell differentiation, extracellular matrix organization, cell adhesion, negative regulation of canonical Wnt signalling, positive regulation of the MAPK cascade, and HF morphogenesis (Tables S16 and S17). KEGG pathway analysis showed that upregulated DEGs were primarily associated with the TGF-beta, cadherin, Hippo, MAPK, and Wnt signalling pathways (Figure 6C; Table S18). By contrast, downregulated DEGs were enriched in the ECM–receptor interaction, focal adhesion, calcium, and FoxO signalling pathways (Figure 6D; Table S19).

### 3.5. Integrative Analysis of CUT&Tag and RNA-Seq to Uncover Key Regulatory Genes Involved in HF Development and Cycling

**Figure 7:**
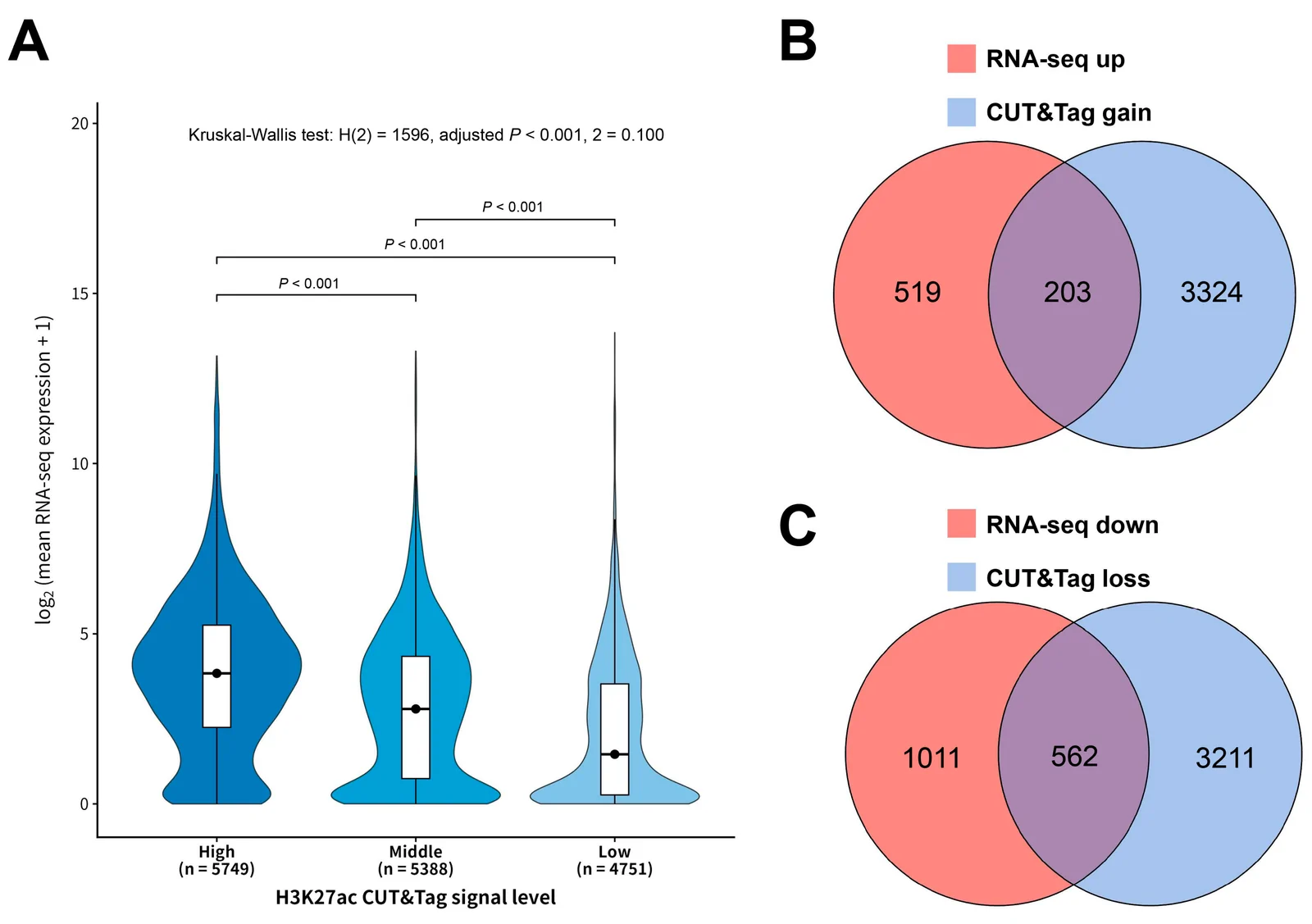
Association between H3K27ac CUT&Tag signal and gene expression across anagen and telogen. (**A**) Violin plots showing RNA-seq expression levels for genes grouped according to H3K27ac CUT&Tag signal levels. Differences among the three groups were assessed using a two-sided Kruskal–Wallis test (H(2) = 1596, *p* < 0.001; ε^2^ = 0.100). Pairwise comparisons were performed using Dunn’s post hoc test with Holm correction for multiple testing. All pairwise comparisons were significant (Holm-adjusted *p* < 0.001). *n* indicates the number of genes. (**B**) Venn diagram showing the overlap between genes associated with H3K27ac gain peaks and upregulated genes. (**C**) Venn diagram showing the overlap between genes associated with H3K27ac loss peaks and downregulated genes. Only DEGs associated with at least one H3K27ac peak were included in the Venn diagrams.

To identify genes potentially associated with H3K27ac-mediated regulatory activity, we integrated the CUT&Tag and RNA-seq datasets and focused on genes showing concordant changes in transcript abundance and H3K27ac enrichment between anagen and telogen. RNA-seq expression differed significantly across the high-, middle-, and low-signal groups (Kruskal–Wallis test, H(2) = 1596, *p* < 0.001; ε^2^ = 0.100). Pairwise Dunn tests, followed by Holm adjustment, showed significant differences for each group comparison (all Holm-adjusted *p* < 0.001). Overall, genes displaying stronger H3K27ac CUT&Tag signals also exhibited greater RNA-seq expression, whereas genes with relatively low expression tended to have less H3K27ac enrichment (Figure 7A). Among the DEGs, 722 of the 1176 upregulated genes and 1573 of the 1966 downregulated genes were associated with at least one H3K27ac peak and were included in the integrative analysis. The integrative analysis identified 765 DEGs that overlapped with genes associated with differentially enriched H3K27ac peaks (Tables S20 and S21). Upregulated DEGs were significantly enriched among H3K27ac-gain-associated genes (203 observed versus 111.26 expected; 1.82-fold enrichment; OR = 2.22, lower 95% confidence bound = 1.92; one-sided Fisher’s exact test, *p* = 6.91 × 10^−19^) (Figure 7B). Similarly, downregulated DEGs were enriched among H3K27ac-loss-associated genes (562 observed versus 253.39 expected; 2.22-fold enrichment; OR = 3.22, lower 95% confidence bound = 2.93; *p* = 9.41 × 10^−87^) (Figure 7C). These concordant changes suggest that differential H3K27ac enrichment is associated with transcriptional differences between the anagen and telogen phases. Specifically, *DLX3*, *GRHL3*, *GRHL1*, *ZNF750*, *TCF7*, *CTNNBIP1*, *VANGL2*, *SMAD7*, and *FGF5* showed increased expression accompanied by enhanced H3K27ac signal. By contrast, *WNT4*, *DKK1*, *DKK2*, *KITLG*, *FGF10*, *BMP6*, *PDGFRA*, *FST*, *TGFB3*, *TGFBR2*, *TGFBR3*, and *SMAD3* showed reduced expression together with decreased H3K27ac enrichment. These genes may represent candidate regulators of HF development and cycling in Tianzhu white yaks.

## 4. Discussion

Histone modifications regulate gene expression by modulating chromatin states and shaping the recruitment of transcription factors and cofactors [14]. Among these modifications, H3K27ac is widely used as a marker of active *cis*-regulatory elements, including promoters and enhancers, and is implicated in biological processes such as cell-cycle progression, cellular differentiation, and apoptosis [37,38,39]. In this study, we used CUT&Tag to profile H3K27ac genome-wide in skin samples from Tianzhu white yaks at the anagen and telogen phases. Integration of CUT&Tag and RNA-seq data revealed stage-associated changes in H3K27ac enrichment and gene expression between anagen and telogen. These changes involved genes and regulatory pathways related to HF development and growth. These findings provide insight into the epigenetic mechanisms underlying stage-specific regulation of the HF cycle in Tianzhu white yaks.

H3K27ac enrichment is well established to correlate positively with transcriptional activity. Previous studies have shown that candidate enhancer regions are typically enriched in H3K27ac and frequently occupied by the histone acetyltransferase p300 [40]. Consistent with this model, genes in the high-H3K27ac-signal group generally exhibited higher expression levels than those in the low-H3K27ac-signal group in both anagen and telogen skin samples. Active enhancers and promoters are generally characterized by increased H3K27ac occupancy [15]. These findings support H3K27ac as a marker of active transcription and suggest that its dynamic changes may contribute to stage-dependent transcriptional regulation between anagen and telogen.

In the present study, we identified 14,496 differentially enriched H3K27ac peaks between anagen and telogen skin samples, including 5618 H3K27ac-gain peaks and 8878 H3K27ac-loss peaks in anagen. These findings indicate substantial stage-dependent remodelling of the active chromatin landscape during the HF cycle. Genomic annotation of the differentially enriched H3K27ac peaks provided additional insight into the regulatory changes associated with the stage-associated differences between anagen and telogen. The proportions of peaks located in distal intergenic regions differed between the H3K27ac-gain and H3K27ac-loss groups. H3K27ac is widely recognized as a marker of active distal regulatory elements. The distribution of differentially enriched peaks suggests that stage-associated chromatin remodelling extends beyond promoter-proximal regions to include putative enhancers. Such distal regulatory elements are thought to play important roles in shaping cell-type-specific and stage-specific transcriptional programmes [41]. In hair follicle stem cells (HFSCs) and their committed progenitors, dynamic remodelling of SEs has been shown to accompany lineage progression and changes in cell-state plasticity, thereby contributing to the regulation of stemness, differentiation, and transcriptional programmes [42]. The distinct genomic distributions of H3K27ac-gain and H3K27ac-loss peaks may reflect different regulatory requirements between anagen and telogen. They also support a potential role for H3K27ac-associated *cis*-regulatory changes in stage-specific transcriptional regulation.

To explore the biological relevance of differential H3K27ac enrichment between anagen and telogen, we performed functional enrichment analyses of genes associated with the differentially enriched H3K27ac peaks. Genes associated with anagen H3K27ac-gain peaks were enriched in pathways closely related to HF development and growth, including the Wnt, BMP/TGF-beta, PI3K-AKT, and MAPK signalling pathways. Among these pathways, Wnt signalling is one of the best-characterized regulators of HF development and is essential for HF induction, maintenance, and activation of HFSCs [43,44]. Wnt signalling promotes the proliferation and differentiation of bulge stem cell toward hair matrix cells, supporting hair shaft production and anagen progression [45]. Increased H3K27ac enrichment at Wnt-related loci in anagen may mark regulatory elements associated with follicular activation and growth. This pattern is consistent with the proliferative and regenerative characteristics of anagen follicles. Previous studies have shown that PI3K-AKT signalling can promote dermal papilla cell proliferation and enhance hair growth, partly through interactions with Wnt/β-catenin signalling [46,47]. Activation of PI3K-AKT signalling enhances the proliferation and survival of hair matrix keratinocytes, whereas impaired PI3K-AKT activity has been associated with premature entry into telogen and follicular regression. Together, these findings suggest that anagen-associated H3K27ac gain is linked to regulatory programmes involved in HFSC activation and follicular proliferation. Conversely, genes associated with H3K27ac-loss peaks in anagen were enriched in signalling pathways implicated in HFSC-state regulation and follicular development, including the JAK-STAT and Hedgehog signalling pathways. Emerging evidence highlights the critical role of JAK-STAT signalling in regulating the hair cycle, particularly in maintaining HFSCs in a quiescent state during telogen under specific physiological conditions. JAK-STAT signalling has been reported to contribute to HFSC quiescence in pregnant and lactating female mice, suggesting that this pathway is modulated by physiological and hormonal conditions to regulate HF activity [48]. Further studies have shown that topical application of small-molecule JAK inhibitors strongly induces anagen initiation in telogen-phase mouse skin [49]. Hedgehog signalling plays essential roles in HF development and postnatal hair cycle regulation. During embryogenesis, Sonic hedgehog (Shh) is required for HF morphogenesis. In adult mouse skin, Hedgehog signalling regulates follicular growth and regeneration, and pharmacological activation of this pathway can promote the transition of telogen follicles into anagen [50]. Taken together, these findings suggest that stage-dependent remodelling of H3K27ac-marked regulatory elements may influence multiple signalling networks involved in HFSC quiescence and follicular reactivation.

Integrative analysis of CUT&Tag and RNA-seq data identified candidate genes involved in HF cycling. Many of the candidate genes identified in this analysis are known to regulate HF growth, regression, and quiescence. Among these genes, FGF5 is a well-established regulator of HF cycling and hair length. In mice, its expression rises toward the end of anagen, while loss of FGF5 function delays catagen induction, prolongs anagen, and results in long-hair phenotype [51]. Studies in farm animals have likewise shown that loss-of-function variants in *FGF5* are associated with prolonged hair growth and long-hair phenotypes, suggesting that its role in controlling hair length is evolutionarily conserved [52,53]. Hair or fleece length depend on both rate of fibre growth and the duration of anagen. During anagen, matrix keratinocytes undergo sustained proliferation and produce the hair shaft; therefore, the duration of this phase is an important determinant of the time available for fibre growth [54]. FGF5 may influence final fleece length by limiting anagen duration and promoting the subsequent transition to catagen, thereby reducing the time available for fibre elongation. In this study, the increased *FGF5* expression and H3K27ac enrichment detected in late-anagen skin are compatible with a possible role for FGF5 in the regulatory processes that precede the anagen-to-catagen transition. PDGF signaling plays an important role in maintaining the homeostasis and function of HF dermal stem cells. A previous study has shown that PDGF/PDGFRA signaling promotes the proliferation and self-renewal of HF dermal stem cells, maintains their cellular populations within the dermal papilla, and promotes subsequent entry into anagen [55]. In the present study, the relatively high expression of PDGFRA during telogen may indicate that PDGFRA-mediated signaling contributes to the maintenance of the dermal stem cell niche during this phase and helps preserve the ability of HFs to re-enter anagen. Overall, these results suggest that stage-associated H3K27ac changes may be related to signalling networks involved in HF growth and phase transitions.

SEs are extended regulatory domains composed of enhancer clusters with exceptionally high chromatin activity [18]. They are typically marked by prominent H3K27ac enrichment and often coincide with dense occupancy by lineage-defining transcription factors and coregulatory complexes [19]. In this study, we applied the ROSE algorithm to genome-wide H3K27ac profiles and identified distinct SE landscapes characteristic of the anagen and telogen phases of the HF cycle. We detected 1119 SEs in anagen and 1074 in telogen, including 310 anagen-specific and 304 telogen-specific SEs. These findings are consistent with a previous study in mouse HFs [56]. They also indicate that the regulatory architecture of the HF cycle is dynamic, with phase-associated transcriptional programmes accompanied by extensive remodelling of SE-associated chromatin domains. SEs may regulate distant target genes through long-range chromatin interactions and chromatin looping. Therefore, the SE-associated genes identified in this study should be interpreted as putative target genes. KEGG pathway analysis revealed that the putative target genes of anagen-specific SEs were significantly enriched in the PI3K-AKT, Notch, and growth hormone synthesis, secretion, and action pathways. These pathways are implicated in progenitor cell proliferation, survival, and differentiation [57,58]. Their enrichment suggests that SE remodelling may contribute to the transcriptional programmes supporting progenitor expansion and follicular morphogenesis during anagen. Notably, the Notch pathway is well established as an essential regulator of HFSC fate [59], whereas the PI3K-AKT pathway is associated with enhanced growth factor responsiveness and cell survival during active follicular growth [46]. In contrast, genes associated with telogen-specific SEs were enriched in the focal adhesion, ECM–receptor interaction, and Rap1 signalling pathways. Their enrichment in telogen-associated SEs may not necessarily indicate full activation of the corresponding transcriptional programmes during telogen, but rather suggest that the corresponding regulatory elements are already established in preparation for the telogen-to-anagen transition [60,61]. Collectively, these data indicate that anagen and telogen are characterized by distinct SE-associated regulatory states corresponding to active follicular growth and stem cell quiescence, respectively. These findings support a model in which stage-specific SEs contribute to the transcriptional plasticity of HF cells and may contribute to transitions between anagen-associated growth and telogen-associated quiescence.

To identify TFs that may participate in H3K27ac-associated regulatory remodelling between anagen and telogen, we performed DNA motif-enrichment analysis using differentially enriched H3K27ac peaks between anagen and telogen. Several of the candidate TFs have established roles in HF biology. LEF1 is a major transcriptional effector of canonical Wnt/β-catenin signaling and is required for HF morphogenesis and the differentiation of HF lineages [62]. TCF3 and LEF1 have been reported to play distinct, context-dependent roles in multipotent skin stem cell fate, with LEF1 promoting Wnt/β-catenin-dependent hair differentiation and TCF3 repressing epidermal terminal differentiation through a largely β-catenin-independent mechanism [63]. LHX2 contributes to the maintenance and growth of HF progenitors and is required for HF morphogenesis and anagen progression, whereas its ectopic expression can induce anagen [64,65]. FOXC1, an early transcription factor expressed during HF development, is required to maintain HFSC quiescence and preserve bulge niche integrity; its loss shortens HFSC quiescence, accelerates HF cycling, and ultimately compromises long-term hair regeneration [66]. As a key transcriptional regulator of epidermal and HF differentiation, GATA3 integrates Notch, Wnt, and BMP signaling to maintain the balance between epidermal and HF cell fates, whereas its loss disrupts HF organization, differentiation, pigmentation, and hair cycling [67]. The enrichment of these motifs in differentially enriched H3K27ac regions between anagen and telogen suggests that the corresponding TFs may participate in regulating stage-dependent transcriptional programmes. However, motif enrichment alone does not establish direct TF binding or activity, and these candidates will require validation through complementary approaches.

HF cycling depends on coordinated interactions between follicular stem cells and their surrounding microenvironment. Our study defines phase-specific H3K27ac-associated regulatory programmes in Tianzhu white yak skin and identifies candidate transcriptional regulators that may contribute to this process. Nevertheless, the regulatory elements and genes identified here provide a foundation for further dissecting the molecular basis of hair-cycle regulation.

However, several limitations should be acknowledged. Samples were classified as anagen or telogen based on previously described seasonal hair-cycle patterns in Tianzhu white yaks, without histological verification of the collected tissues. In addition, primary and secondary HFs were neither separately identified nor measured; therefore, their relative proportions in the analyzed tissue are unknown. Accordingly, the observed molecular differences should be interpreted as group-level differences between samples seasonally as anagen and telogen. Although these differences are consistent with hair-cycle-associated regulation, they cannot be conclusively attributed to a specific primary- or secondary-follicle category without direct histological staging and quantitative assessment of follicle types.

A further limitation is that CUT&Tag and RNA-seq were performed on bulk, full-thickness skin samples. HF cycling is accompanied by extensive tissue remodelling and changes in the abundance and activation states of multiple cell populations. Accordingly, the differences in H3K27ac enrichment and gene expression between anagen and telogen may reflect both changes in cellular composition and cell-intrinsic regulation. For example, the telogen-associated enrichment of extracellular matrix, focal adhesion, and connective-tissue pathways may partly arise from differences in the abundance of dermal papilla cells, upper follicle-associated cells, inner root sheath-associated cells, and other skin populations. These signals may not exclusively represent regulation within an individual follicular cell type. This interpretation is consistent with single-cell transcriptomic studies showing pronounced cellular heterogeneity and hair-cycle-associated changes in cell-population proportions in yak skin [11]. However, the current dataset does not allow us to resolve the cellular sources of the observed molecular changes or to distinguish compositional variation from cell-intrinsic regulatory effects. Future studies combining cell-type-resolved profiling and spatial multi-omics will be required to clarify the cellular basis of hair-cycle-associated molecular variation.

## 5. Conclusions

In conclusion, genome-wide profiling of H3K27ac in skin tissues from Tianzhu white yaks during the anagen and telogen phases revealed a distinct phase-specific chromatin landscape associated with HF cycling. Functional analyses of genes associated with differentially enriched H3K27ac regions and stage-specific SEs implicated these regulatory elements in biological processes relevant to HF development and cycling. Regulatory-region analyses further identified LEF1, LHX2, FOXN1, and GATA3 as candidate transcriptional regulators associated with stage-specific follicular regulatory programmes. Together, these findings provide insights into the epigenetic regulation of the hair cycle in Tianzhu white yak and establish a foundation for future studies of the molecular mechanisms underlying hair growth and cycling.

## Data Availability

The original data presented in the study are openly available in the National Genomics Data Center (CNCB) under accession number PRJCA072809.

## Supplementary Materials

The following supporting information can be downloaded at: https://www.mdpi.com/article/10.3390/biology15211920/s1, Figure S1: Principal component analysis of ATAC-seq data; Table S1: Summary of CUT&Tag data; Table S2: Annotation of H3K27ac peaks in skin during anagen and telogen phases; Table S3: Annotation of differentially enriched H3K27ac peaks in skin between anagen and telogen phases; Table S4: Significantly enriched GO terms for genes associated with H3K27ac-gain peaks during anagen; Table S5: Significantly enriched KEGG pathways for genes associated with H3K27ac-gain peaks during anagen; Table S6: Significantly enriched GO terms for genes associated with H3K27ac-loss peaks during anagen; Table S7: Significantly enriched KEGG pathways for genes associated with H3K27ac-loss peaks during anagen; Table S8: Motifs enriched in differentially enriched H3K27ac peaks; Table S9: Ranked super-enhancers and their annotations in the anagen phase of skin; Table S10: Ranked super-enhancers and their annotations in the telogen phase of skin; Table S11: Significantly enriched BP terms for putative target genes of anagen-specific super-enhancer; Table S12: Significantly enriched GO terms for putative target genes of telogen-specific super-enhancer; Table S13: Significantly enriched KEGG pathways for putative target genes of anagen-specific super-enhancer; Table S14: Significantly enriched KEGG pathways for putative target genes of telogen-specific super-enhancer; Table S15: Differentially expressed genes in skin between anagen and telogen phases; Table S16: Significantly enriched GO terms for DEGs upregulated in anagen; Table S17: Significantly enriched GO terms for DEGs downregulated in anagen; Table S18: Significantly enriched KEGG pathways for upregulated DEGs in anagen; Table S19: Significantly enriched KEGG pathways for downregulated DEGs in anagen. Table S20. Intersection of upregulated genes and genes associated with H3K27ac gain peaks in the anagen phase. Table S21. Intersection of downregulated genes and genes associated with H3K27ac loss peaks in the anagen phase.
